# Establishment and characterization of 6 novel patient-derived primary pancreatic ductal adenocarcinoma cell lines from Korean pancreatic cancer patients

**DOI:** 10.1186/s12935-017-0416-8

**Published:** 2017-04-20

**Authors:** Mi-Ju Kim, Min-Sun Kim, Sung Joo Kim, Soyeon An, Jin Park, Hosub Park, Jae Hoon Lee, Ki-Byung Song, Dae Wook Hwang, Suhwan Chang, Kyu-pyo Kim, Seong-Yun Jeong, Song Cheol Kim, Seung-Mo Hong

**Affiliations:** 1 Asan Institute for Life Sciences, Asan Medical Center, University of Ulsan College of Medicine, Seoul, South Korea; 2 Department of Pathology, Asan Medical Center, University of Ulsan College of Medicine, 88, Olympic-ro 43-gil, Songpa-gu, Seoul, 05505 Republic of Korea; 3 Department of Surgery, Asan Medical Center, University of Ulsan College of Medicine, 88, Olympic-ro 43-gil, Songpa-gu, Seoul, 05505 Republic of Korea; 4 Department of Biomedical Sciences, University of Ulsan College of Medicine, Seoul, South Korea; 5 Department of Physiology, University of Ulsan College of Medicine, Seoul, South Korea; 6 Department of Oncology, Asan Medical Center, University of Ulsan College of Medicine, Seoul, South Korea; 7 Center for Advancing Cancer Therapeutics, Asan Medical Center, University of Ulsan College of Medicine, Seoul, South Korea

**Keywords:** Pancreas, Cancer, Ductal adenocarcinoma, Primary, Cell line

## Abstract

**Background:**

Pancreatic ductal adenocarcinomas are among the most malignant neoplasms and have very poor prognosis. Our understanding of various cancers has recently improved the survival of patients with cancer, except for pancreatic cancers. Establishment of primary cancer cell lines of pancreatic ductal adenocarcinomas will be useful for understanding the molecular mechanisms of this disease.

**Methods:**

Eighty-one surgically resected pancreatic ductal adenocarcinomas were collected. Six novel pancreatic cancer cell lines, AMCPAC01–06, were established and histogenetic characteristics were compared with their matched tissues. The clinicopathologic and molecular characteristics of the cell lines were investigated by *KRAS* and *TP53* sequencing or SMAD4 and p53 immunohistochemistry. Xenografts using AMCPAC cell lines were established.

**Results:**

From the 81 pancreatic ductal adenocarcinomas, six (7.4% success rate) patient-derived primary cell lines were established. The six AMCPAC cell lines showed various morphologies and exhibited a wide range of doubling times. AMCPAC cell lines contained mutant *KRAS* in codons 12, 13, or 61 and *TP53* in exon 5 as well as showed aberrant p53 (5 overexpression and 1 total loss) or DPC4 (all 6 intact) expression. AMCPAC cell lines demonstrated homology for the *KRAS* mutation and p53 expression compared with matched primary cancer tissues, but showed heterogeneous DPC4 expression patterns.

**Conclusions:**

The novel AMCPAC01–06 cell lines established in this study may contribute to the understanding of pancreatic ductal adenocarcinomas.

*Trial registration* Retrospectively registered

**Electronic supplementary material:**

The online version of this article (doi:10.1186/s12935-017-0416-8) contains supplementary material, which is available to authorized users.

## Background

Ductal adenocarcinomas are the most common malignant neoplasms of the pancreas and account for approximately 85–90% of malignant neoplasms arising from the pancreas (known as pancreatic cancers). Pancreatic cancers are the ninth most common cancer in Korea [1], and the 5-year survival rate of patients with this type of cancer is only 9% [1]. Surgical resection is the main stay for treating pancreatic cancer, but most patients are inoperable at the time of diagnosis, and only 30% of patients can undergo surgical resection [2]. Approximately 80% of patients show local recurrence or distant metastasis after surgical removal of tumors [2]. Other treatments, such as chemotherapies, are required to improve the survival time of patients with pancreatic cancer [3–6]. However, currently used chemotherapeutic regimens fail to significantly improve the survival time of these patients [7–9]. Therefore, development of new therapeutic modalities is important for improving the survival time of pancreatic cancer patients.

Recently, several trials were conducted to examine customized cancer treatment using patient-derived pancreatic cancer tissues [10–13]. However, using surgically resected pancreatic cancer tissue is difficult because of the limited amount of residual pancreatic cancer tissues after the submission of large amount of cancer tissues for pathologic examination for precise diagnosis and staging. To overcome this limitation, researchers have attempted to establish cancer cell lines from patient-derived cancer tissues and use various molecular pathologic studies with these cancer cell lines for tailored patient treatments. However, it is difficult to establish patient-derived primary cancer cell lines from pancreatic cancers because of specific histopathologic characteristics of pancreatic cancer such as low cancer cellularity of pancreatic cancer, and the occurrence of extensive desmoplastic reactions by overproduction of cancer-associated fibroblasts. Thus, the number of established patient-derived pancreatic cancer cell lines is currently much lower than that of cancers from other organs. At present, 21 pancreatic cancer cell lines have been established, including 11 cell lines from American Type Culture Collection and 3 cell lines form Korean Cell Line Bank [14–16].

In addition, some different clinicopathologic features were observed in pancreatic cancer patients of different ethnicities. Therefore, establishing novel primary pancreatic cancer cell lines derived from Korean pancreatic cancer patients and applying various chemotherapeutic regimens to the developed primary pancreatic cancer cell lines can help in the determination of highly sensitive chemotherapeutic regimens for individual pancreatic cancer patients, particularly when regional recurrence or distant metastasis develops.

## Methods

### Specimen collection

After approval (2015-0480) from the institutional review board, fresh tumor tissues measuring 0.5 × 0.5 × 0.2 cm^3^ in size were obtained from 81 surgically resected pancreatic ductal adenocarcinomas, immediately soaked in RPMI640 media (Sigma-Aldrich Corp., St. Louis, MO, USA), and transferred in a laminar flow biosafety cabinet.

### In vitro cell culture

Pancreatic tumor tissues were rinsed with Hank’s balanced salt solution in clean bench, minced into <2 mm^3^ fragments, and digested with 0.1% (W/V) of collagenase type 1 (GIBCO, Grand Island, NY, USA) at 37 °C for 20 min. The resulting fragments were centrifuged at 200×*g* for 5 min, washed thrice with phosphate-buffered saline, plated onto RPMI1640 media (GIBCO) containing 10% fetal bovine serum (GIBCO) and 1% penicillin/streptomycin (GIBCO), and allowed to adhere. After incubation for several days, mixed growth of cancer cells and fibroblasts was observed in the tissue fragments. To overcome fibroblast overgrowth, periodic trypsinization was conducted by incubation with 0.005% trypsin/EDTA (GIBCO) at 37 °C for 3 min during 2–3 passages to remove fibroblasts, and unwanted fibroblasts were detached by pipetting. The primary cell culture was monitored with a phase-contrast microscope. Cancer cells were grown at 37 °C in a humidified atmosphere with 5% CO_2_.

### Growth rate analysis of established cell lines

The cell growth rate was measured using 3-(4,5-dimethylthiazol-2-yl)-2,5-diphenyltetrazolium bromide (MTT, Sigma-Aldrich) at 24-h intervals. After 1 × 10^4^ cells were seeded into 96-well plates, 0.5 mg/mL MTT was added over consecutive days for violet pellet formation by living cells. The pellets were solubilized in 200 μL of dimethyl sulfoxide. The optical density of each sample was measured at 570 nm using a microplate reader (Sunrise Reader, Tecan, Männedorf, Switzerland). Growth rate was measured as a percentage of control growth. Cells from passage 15 were used to determine population doubling time, and all experiments were repeated twice in triplicate.

### Characterization of cell lines

#### Construction cell microarray

After fixation of 5 × 10^6^ cancer cells with a Cytorich Red fixative solution (BD Biosciences, Franklin Lakes, NJ, USA) for 48 h, the supernatant was removed after centrifugation. The pellets were additionally fixed with 95% ethanol for 60 min then embedded in paraffin. Each cancer cell block was selected as a donor, and the designated areas for each cell block were punched with a 5-mm diameter cylinder by a Manual Tissue Microarrayer (Uni TMA Co., Ltd., Seoul, Korea) and transferred to a recipient block, and cell microarrays (CMAs) were constructed.

#### Immunohistochemistry

Immunohistochemical labeling was performed by the immunohistochemical laboratory of the Department of Pathology, Asan Medical Center. Briefly, 4-μm tissue sections from the CMA and matched formalin-fixed paraffin-embedded (FFPE) primary cancer tissues of ductal adenocarcinomas were deparaffinized and hydrated in xylene and serially diluted with ethanol, respectively. Endogenous peroxidase was blocked by incubation in 3% H_2_O_2_ for 10 min, and heat-induced antigen retrieval was performed. Primary antibodies with Benchmark autostainer (Ventana Medical Systems, Tucson, AZ, USA) were used as per the manufacturer’s protocol. Primary antibodies for cytokeratin 19 (clone A53-B/A2.26; 1:200; Cell Marque, CA, USA), p53 (clone DO-7; 1:3000; DAKO, Glostrup, Denmark), and DPC4 (clone EP618Y, 1:100; GeneTex, Irvine, CA, USA) were incubated at room temperature for 32 min, and the sections were labeled with an automated immunostaining system with the I-View detection kit (Benchmark XT; Ventana Medical Systems). Immunostained sections were lightly counterstained with hematoxylin, dehydrated in ethanol, and cleared in xylene.

### Detection of *TP53* and *KRAS* mutations

The genomic DNA of the established cell lines was extracted using the QIAamp DNA Micro kit (Qiagen, Hilden, Germany) following the manufacturer’s protocol. Polymerase chain reaction (PCR) amplification was performed with 10 ng of DNA covering exons 5–8 of the *TP53* gene with intragenic primers flanking these exons as previously described [17]. PCR-amplified products were purified using a QIAquick column (Qiagen). *TP53* gene sequencing was performed with BigDye 3.1 and a 3730xl DNA analyzer (Applied Biosystems, Foster City, CA, USA).

Similarly, pyrosequencing was performed to detect *KRAS* at codons 12, 13, and 61. Primer sequences of *KRAS* are summarized in Table 1. DNA (10 ng) was amplified using a biotin-labeled primer covering codons 12, 13, and 61 of *KRAS*. The biotin-labeled PCR products were immobilized on Streptavidin Sepharose HP beads (GE Healthcare, Little Chalfont, UK) and the immobilized PCR products were sequenced using a pyrosequencer, PyroMark Q96MD System (Qiagen) according to the manufacturer’s protocol. Primer sequences used to amplify *TP53* and *KRAS* are shown in Table 1.

**Table 1:** Primer sequences and PCR conditions of *TP53* and *KRAS*

Target	Forward primer	Reverse primer	AT (°C)	Size (bp)
*TP53* exon 5	5′-CACTTGTGCCCTGACTTTCA-3′	5′-AACCAGCCCTGTCGTCTCT-3′	64	267
*TP53* exon 6	5′-CAGGCCTCTGATTCCTCACT-3′	5′-CTTAACCCCTCCTCCCAGAG-3′	64	185
*TP53* exon 7	5′-CCACAGGTCTCCCCAAGG-3′	5′-CCAGGTCAGGAGCCACTT-3′	64	179
*TP53* exon 8	5′-GCCTCTTGCTTCTCTTTTCC-3′	5′-TAACTGCACCCTTGGTCTCC-3′	62	217
*KRAS* 12 and 13	5′-GGTGAGTTTGTATTAAAAGGTACTGG-3′	5′-Biotin-GCTGTATCGTCAAGGCACTCTT-3′	56	100
*KRAS* 61	5′-TGGAGAAACCTGTCTCTTGGATAT-3′	5′-Biotin-TACTGGTCCCTCATTGCA CTGTA-3′	60	72

*AT* annealing temperature

### In vivo tumorigenicity by tumor xenograft

Tumorigenicity in mice was confirmed by subcutaneous injection of approximately 2 × 10^6^ cancer cells from culture bilaterally into each flank of male NSG (5 weeks old) mice. Xenograft tumor growth was monitored twice per week for 3 months. Tumor volume (TV) was calculated according to the formula: TV (mm^3^) = length × width^2^ × 0.5. When a tumor size of 100–200 mm^3^ was reached, tumors were explanted from the mice. The explanted tumor xenografts were used for reimplantation and FFPE to confirm the histology.

### Statistical analysis

The significance of differences between experimental conditions was determined using the student’s *t* test and Mann–Whitney U test for unpaired observations.

## Results

### Establishment of pancreatic cancer cell lines

Fresh tissues from 81 patients diagnosed with pancreatic ductal adenocarcinomas were used to establish primary cancer cell lines. Among the 81 cases, 75 cases (93%) failed to establish cell lines because of fungal or mycoplasmal contamination in 30 cases (37%) and overgrowth of cancer-associated fibroblasts in 45 cases (56%). Six (7.4%) patient-derived pancreatic cancer cell lines were established and named, AMCPAC01–AMCPAC06. The first sub-culture was performed 7–15 days after initiating primary culture for attachment and spreading, and the AMCPAC cell lines were passed a minimum of 10 times.

### Clinicopathologic characteristics of cancer cell lines

**Fig. 1:**
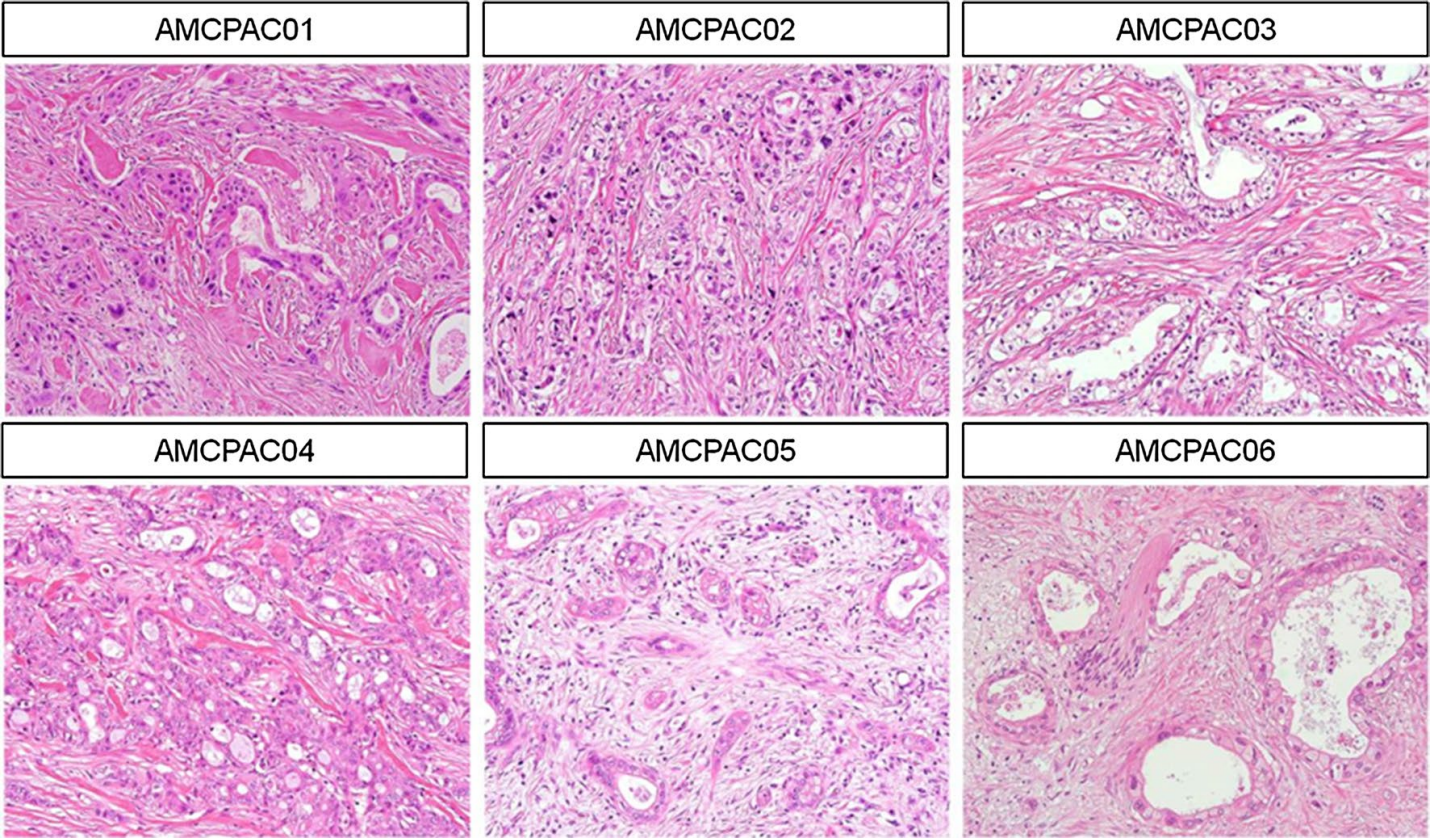
Representative hematoxylin and eosin staining images of FFPE pancreatic cancer tissue

Clinicopathologic characteristics of the established cancer cell lines are summarized in Table 2. Briefly, cell lines were derived from five males and one female. The mean age of the patients was 52.0 ± 12.1 years (range 30–67 years). The pathologic diagnosis of all six cases was moderately differentiated ductal adenocarcinoma (Fig. 1). Tumor locations were as follows: tail of the pancreas in four cases, body in 1 case, and head in 1 case. The mean tumor size was 3.6 ± 1.8 cm (range 2.1–6.9 cm). Five cases showed peripancreatic soft tissue extension, while one case invaded the surrounding organs, spleen, and transverse colon (AMCPAC04). Lymphovascular invasions were identified in 3 cases (AMCPAC02, AMCPAC04, and AMCPAC05) and perineural invasions were observed in 5 cases, except for case #6. Lymph node metastases were detected in 3 cases (AMCPAC03, AMCPAC04, and AMCPAC06).

**Table 2:** Clinicopathologic characteristics of established cancer cell lines

Clinicopathologic factors	AMCPAC01	AMCPAC02	AMCPAC03	AMCPAC04	AMCPAC05	AMCPAC06
Age (years)	30	67	56	53	52	54
Sex	Male	Female	Male	Male	Male	Male
Operation name	DP	DP	DP	DP	PPPD	DP
Pathologic diagnosis	Ductal adenocarcinoma	Ductal adenocarcinoma	Ductal adenocarcinoma	Ductal adenocarcinoma	Ductal adenocarcinoma	Ductal adenocarcinoma
Differentiation	MD	MD	MD	MD	MD	MD
Location	Body	Tail	Tail	Tail	Head	Tail
Tumor size (cm)	2.1	2.5	4.5	6.9	2.8	2.9
pT classification	pT3	pT3	pT3	pT4	pT3	pT3
pN classification	pN0	pN0	pN1	pN1	pN0	pN1
Lymphovascular invasion	Absent	Present	Absent	Present	Present	Absent
Perineural invasion	Present	Present	Present	Present	Present	Absent

*DP* distal pancreatectomy, *PPPD* pylorus preserving pancreatecticoduodenectomy, *MD* moderately differentiated

### Cytological characteristics of cancer cells

**Fig. 2:**
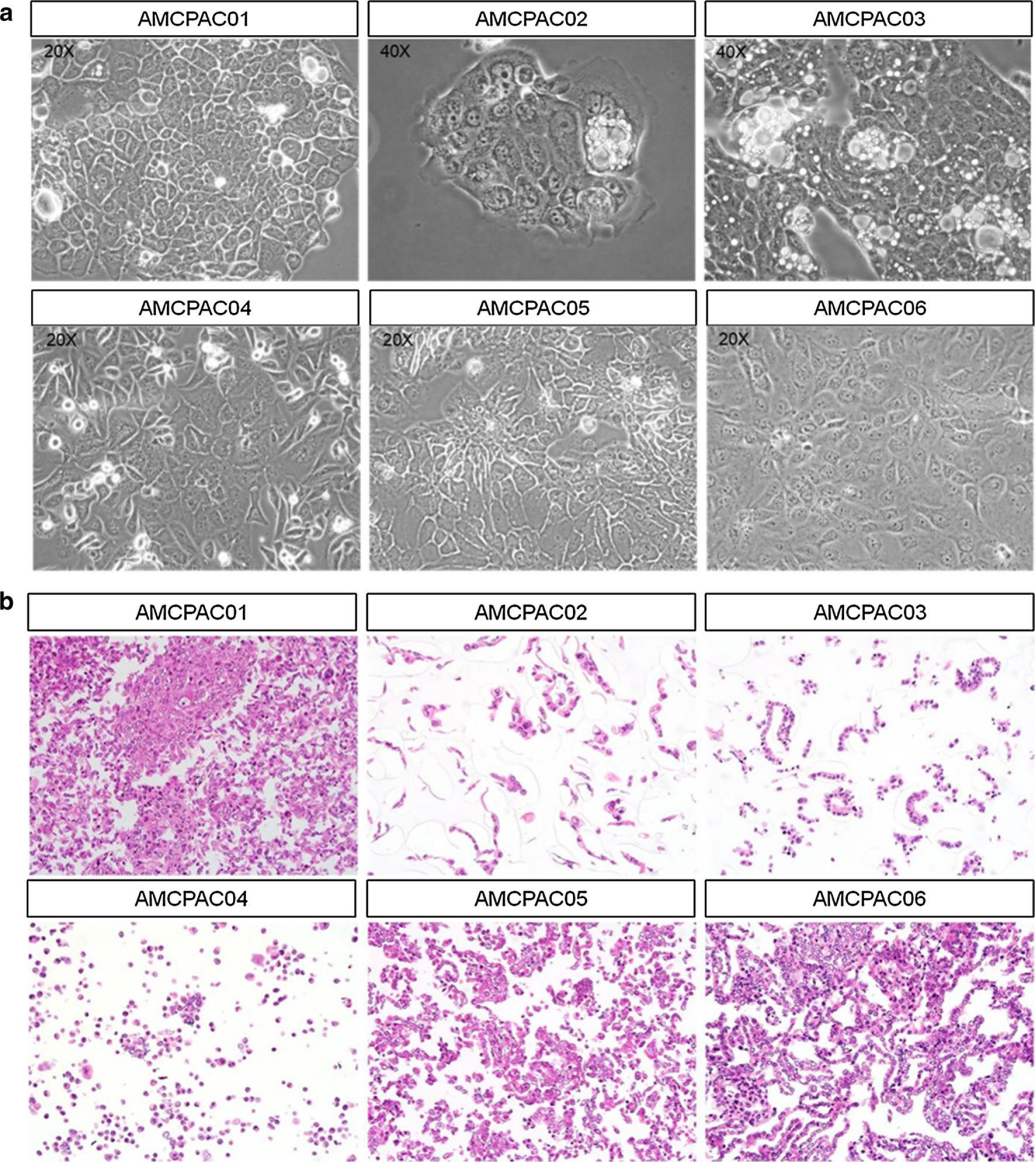
Representative AMCPAC cell line images. **a** Morphology of AMCPAC01–AMCPAC06 cell lines. **b** H&E staining images of AMCPAC01–AMCPAC06 (all, ×20)

**Fig. 3:**
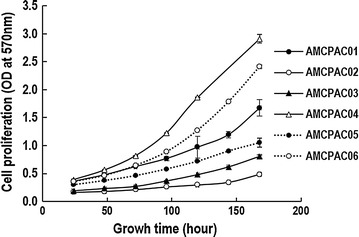
Growth curve of AMCPAC cell lines as determined by MTT assay. Cell proliferation represented by optical density (OD) at 570 nm was measured during seven days. Each value represents the mean ± SE of triplicate determinants

To more efficiently investigate cytological characteristics, CMAs were constructed with the AMCPAC cell lines. The CMA block was composed of 6 wells containing each AMCPAC cell line, and CMA sections were stained with hematoxylin and eosin; matched tissue samples of pancreatic ductal adenocarcinomas were included (Fig. 2b). Cytological evaluation revealed that all six cancer cell lines were adenocarcinomas with mucin production and monolayer growth on the culture dish surface (Fig. 2a). Cytologically, all 6 cancer cell lines showed round to oval (AMCPAC01, AMCPAC02, AMCPAC03, AMCPAC05, and AMCPAC06) or polygonal (AMCPAC04) shapes. The population doubling time of cancer cells was measured by MTT assay. The growth rates of AMCPAC04 and AMCPAC06 were rapid (doubling time of approximately 2 days), while those of AMCPAC02, AMCPAC03, and AMCPAC05 were relatively slow (approximately 3–4 days) based on 24-h growth after cell seeding (Table 3; Fig. 3).

**Table 3:** Cytologic characteristics and growth rate of established cell lines

Cell line	Growth characteristics	Cell morphology	Population doubling time (h)
AMCPAC01	Adherent	Round/oval	68
AMCPAC02	Adherent	Round/oval	87
AMCPAC03	Adherent	Round/oval	68
AMCPAC04	Adherent	Polygonal	48
AMCPAC05	Adherent	Round/oval	75
AMCPAC06	Adherent	Round/oval	51

### Mutational and immunohistochemical status of cancer cell lines

**Fig. 4:**
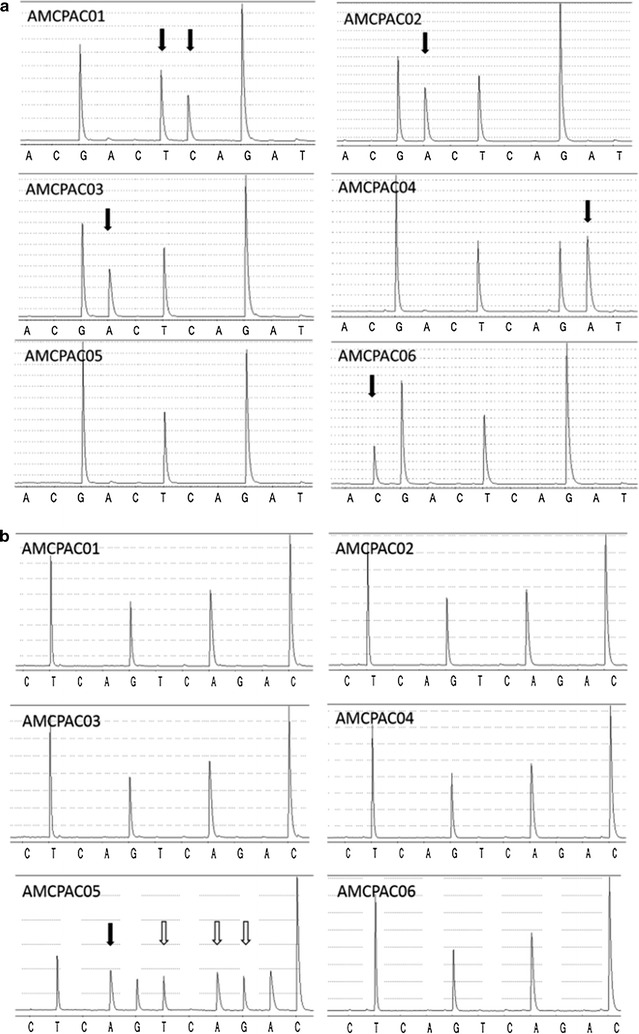
Representative pyrogram images of *KRAS* sequencing **a** of codons 12 and 13 and **b** codons 60 and 61. Different cell lines harbor different *KRAS* gene mutations. **a** Various missense mutations (*black arrow*) are noted in codons 12 and 13 of AMCPAC cell lines; G12V (GGT → GTC) in AMCPAC01, G12D (GGT → GAT) in AMCPAC02 and AMCPAC03, G13D (GGC → GAC) in AMCPAC04, and AMCPAC06 G12R (GGT → CGT). Only AMCPAC05 shows no mutation in codon 12 and 13 (GGTGGC), but codon 60 and 61 mutation of AMCPCA cell lines **b**. In codons 60–61, GGTCAA sequence assays in reverse orientation as TTGCAA. Only AMCPAC05 has 2 mutations; insertion (*white arrow*) and missense mutation (*black arrow*) (GGTCAA → GGT*CTA*CTA). While other cell lines show wile type *KRAS*. *Arrows* indicate *KRAS* mutation site in each cell lines, and among them *white arrow* is newly founded mutated site in this study

Mutant *KRAS* was detected in all AMCPAC cell lines. Mutations in *KRAS* codon 12 were detected in AMCPAC01, AMCPA02, AMCPA03, and AMCPA06, while a mutation in *KRAS* codon 13 was detected in AMCPAC04. And two mutations in *KRAS* codons 60 and 61 were detected in AMCPAC05 (Table 4). Representative images of *KRAS* mutations are depicted in Fig. 4.

**Table 4:** *KRAS* mutation analysis in AMCPAC cell lines

Cell line	c. Description	Codon number	Protein description	Mutation type
AMCPAC01	c.35_36GT>TC^a^	12	p.Gly12Val(G12V)	Missense
AMCPAC02	c.35G>A^a^	12	p.Gly12Asp(G12D)	Missense
AMCPAC03	c.35G>A^a^	12	p.Gly12Asp(G12D)	Missense
AMCPAC04	c.38G>A^a^	13	p.Gly13Asp(G13D)	Missense
AMCPAC05	c.180_181insCTA	60, 61	p.Gly60_Gln61insLeu	Insertion
AMCPAC05	c.182A>T^a^	61	p.Gln61Leu(Q61L)	Missense
AMCPAC06	c.34G>C^a^	12	p.Gly12Arg(G12R)	Missense

*NA* not applicable

^a^Pathogenic

Sequencing analysis of *TP53* revealed the deletion of exon 5 in AMPCPAC01 and missense mutations in 3 lines (AMCPAC04, AMCPAC05, and AMCPAC06) based on Clinvar database analysis (Table 5, Additional file 1: Figure S1).

**Table 5:** Exon 5 on *TP53* mutation in AMCPAC cell lines

Cell line	c. Description	Codon number	Protein description	Mutation type
AMCPAC01	c.384_393del10	128–131	N.D	Deletion
AMCPAC02	N.A	N.A	N.A	Wild-type
AMCPAC03	N.A	N.A	N.A	Wild-type
AMCPAC04	c.380C>T^a^	127	p.S127F	Missense
AMCPAC05	c.398T>A	133	p.M133K	Missense
AMCPAC06	c.451C>T^b^	151	p.P151S	Missense

*NA* not applicable, *N.D* not detectable

^a^Likely pathogenic

^b^Pathogenic

**Fig. 5:**
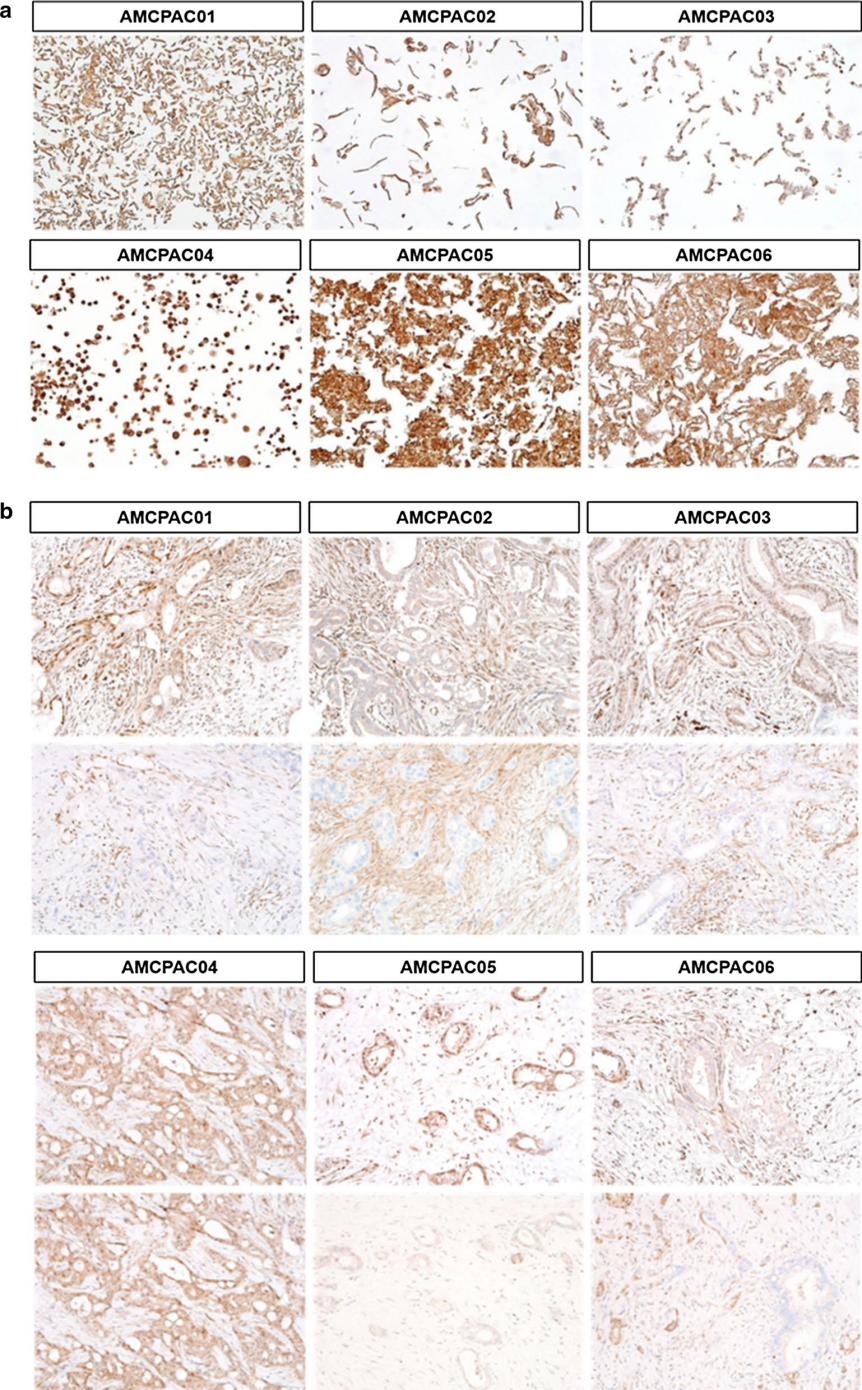
Heterogeneous DPC4 expression within original cancer tissues compared with AMCPAC cell line. **a** DPC4 expression in AMCPAC cell lines. **b** DPC4 expression in cancer tissues

**Fig. 6:**
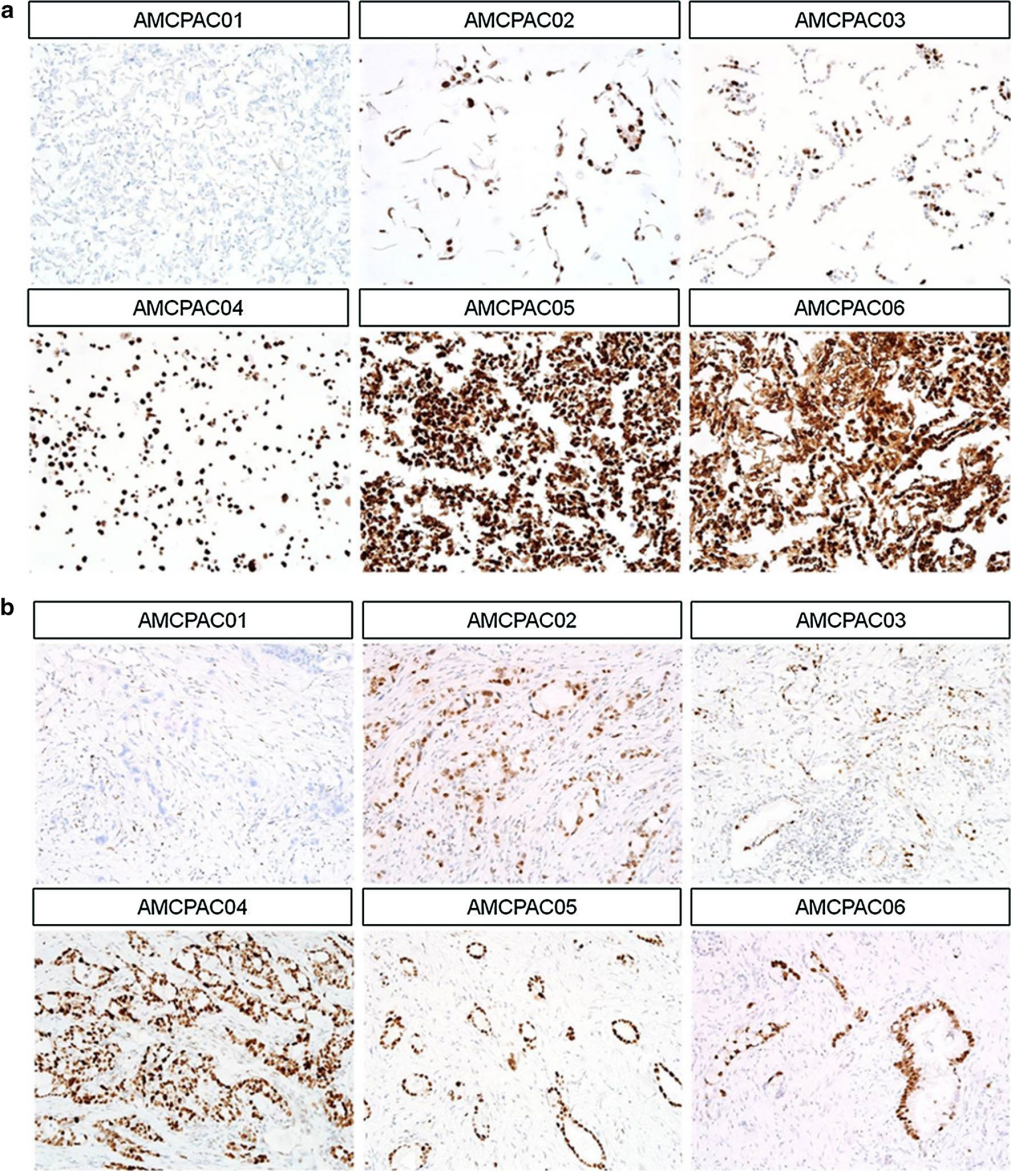
Representative p53 immunohistochemical staining. **a** p53 expression in AMCPAC cells lines. **b** p53 expression in cancer tissues

Representative images of DPC4 and p53 immunolabeling are shown in Figs. 5 and 6 and summarized in Table 6. All 6 cancer cell lines showed intact DPC4 labeling (Fig. 5a). Overexpression of p53 protein was observed in 5 cancer cell lines (AMCPAC02–06; Fig. 6a), while 1 cell line showed a total loss of p53 expression (AMCPAC01). All 6 cases showed matched p53 expression patterns between pancreatic cancer tissue and primary cell lines (Fig. 6b). However, only AMCPAC04 showed matched DPC4 expression between the primary tumor and cancer cell line, while the other 5 tissues showed heterogeneous expression of DPC4 (Fig. 5b).

**Table 6:** Summary of DPC4 and p53 immunohistochemistry staining in AMCPAC cell lines and matched cancer tissues

Cell line	Primary cancer cell line		Cancer tissue	
	DPC4	P53	DPC4	P53
AMCPAC01	Intact	Total loss	Heterogeneous (intact/loss)	Total loss
AMCPAC02	Intact	Overexpression	Heterogeneous (intact/loss)	Overexpression
AMCPAC03	Intact	Overexpression	Heterogeneous (intact/loss)	Overexpression
AMCPAC04	Intact	Overexpression	Intact	Overexpression
AMCPAC05	Intact	Overexpression	Heterogeneous (intact/loss)	Overexpression
AMCPAC06	Intact	Overexpression	Heterogeneous (intact/loss)	Overexpression

### Tumorigenicity of AMCPAC cell lines in immunodeficient mice

**Fig. 7:**
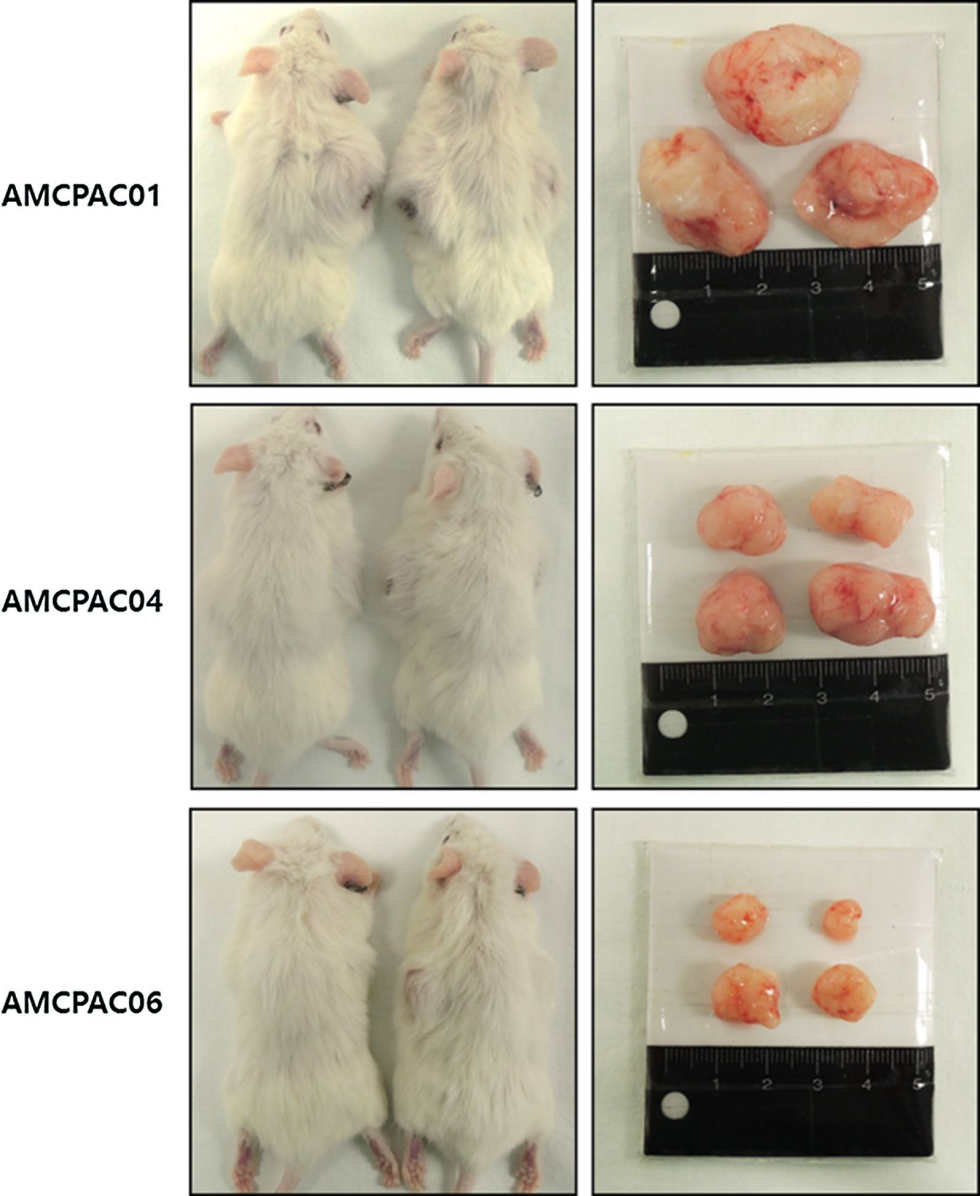
Construction of xenograft of AMCPAC cell lines

AMCPAC cell lines were implanted into 12 mice to test in vivo tumorigenicity. Three months after injection, the AMCPAC01, AMPCPAC04, and AMCPAC06 cell lines were developed, while AMCPAC02, AMPCPAC03, and AMPCPAC05 did not reach a sufficient tumor mass size for extraction from mice. AMCPAC01, AMCPAC04, and AMCPAC06 tumors collected from mice were 100–200 mm^2^ in diameter and used for construction of xenograft cell lines or xenograft FFPE tissue blocks for further analysis (Fig. 7).

## Discussion

In the present study, we established 6 novel patient-derived primary cancer cell lines (AMCPAC cell lines) from Korean patients with pancreatic ductal adenocarcinomas. The success rate of cancer cell line establishment was 7.4%. The main reason for failure was overgrowth of cancer-associated fibroblasts, accounting for 56% of samples. Ductal adenocarcinoma of the pancreas is characterized by extensive desmoplastic stromal reactions; therefore, the density of cancer cells in limited areas was very low. This low cellularity of cancer cells may prohibit the establishment of primary pancreatic cancer cell lines. To overcome these limitations, several primary cell culture methods have been developed such as patient-derived tumor xenografts or three-dimensional tumor organoid models [18–24]. In this study, we modified basic and rapid primary cell culture protocols based on the different reaction rates of trypsin-induced cell dissociation effects depending on cell type. This primary cell culture protocol did not show the expected success rate, but did select pure cancer cells.

*KRAS* is the most commonly activated oncogene in pancreatic cancer. More than 90% of pancreatic cancer patients show *KRAS* mutations, including a low grade of pancreatic intraepithelial neoplasm, a pancreatic cancer precursor [25, 26]. In Korean pancreatic cancer patients, 54% *KRAS* mutations were reported to commonly occur as G12D (GGT → GAT, 31%) and G12 V (GGT → GTT, 34%) on codon 12 by Sanger sequencing [27]. In the present study, *KRAS* mutations were evaluated by pyrosequencing, which is more sensitive compared to conventional Sanger sequencing. All 6 pancreatic cell lines contained various *KRAS* mutations, 4 of them had mutation in codon 12, one in codon 13, which was observed in previous whole exome sequencing studies [28–30]. The G12D, G12R, and G13D *KRAS* mutations detected in AMCPAC cell lines were reported in various cancers, such as pancreatic carcinoma, non-small cell lung cancer, stomach cancer, colorectal cancer, and leukemia [31–36]. Nucleotide exchanges (GGT → GTC) encoding the G12 V *KRAS* mutation in AMCPAC01 are rare, but have been previously reported in one pancreatic and one colorectal cancer patients [37, 38]. In AMCPAC05, two distinct *KRAS* mutations were detected throughout proximal region of codon 61. Q61L *KRAS* mutation is also rarely reported in pancreatic cancer or neoplasm [39, 40]. However, insertion of leucine between codon 60 and 61 (p.Gly60_Gln61insLeu) on *KRAS* has not reported any previous studies and variation databases, including ClinVar and COSMIC (catalogue of somatic mutations in cancer). Finally, AMCPAC cell lines harbor various and noble *KRAS* mutations and these are useful resources for pancreatic cancer research when associated with *KRAS* mutation type.

The mutational status of *TP53* was analyzed because genetic alternations in *KRAS*, *TP53*, and *SMAD4* play key roles in the tumorigenesis of pancreatic ductal adenocarcinoma [41–43]. *TP53* mutations are frequently mutated in pancreatic cancer; approximately 38% of Korean pancreatic cancer patients contain *TP53* alternations [27]. Four AMCPAC cell lines contained *TP53* mutations only in exon 5, and AMCPAC02 and AMCPAC03 showed no *TP53* gene alterations. *TP53* mutations in AMCPAC04 and AMCPAC06 cells are known pathogenic mutations, while those in AMCPAC01 and AMCPAC05 were newly identified [44].

CMAs are novel multiplex array techniques for immunocytochemistry evaluation to detect the molecular composition and function of cell lines. The use of an agarose matrix or agarose mold for cell microarray construction has been reported. In this study, a cell block was used for cell microarray by directly paraffin embedding the cell pellet, resulting in the highest cell density in an array core compared to other methods [45].

It is known that *KRAS*, *p16/DCKN2A*, *GNAS*, and *BRAF* are mutated early in pancreatic cancer progression, while *SMAD4/DPC4* and *TP53* are mutated at later stages [25, 46]. To compare DPC4 and p53 expression levels between established cell lines and matched cancer tissues, immunocytochemistry was performed on CMA sections containing 6 AMCPAC cell lines and matched cancer tissues. Five cells lines, AMCPAC02–06, and matched cancer tissues showed p53 protein overexpression, while AMCPAC01 showed a total loss of p53 expression. A lack of p53 expression is associated with a nonsense mutation (or null mutation) in *TP53* [47]. Our *TP53* sequencing revealed a deletion (TGCCCTCAAC) on exon 5 of *TP53* which was associated with the loss of p53 protein expression. Several studies reported worse prognosis of cancer patients with a lack of p53 expression compared to those with missense *TP53* mutations in lung and ovarian cancers [48, 49].

**Fig. 8:**
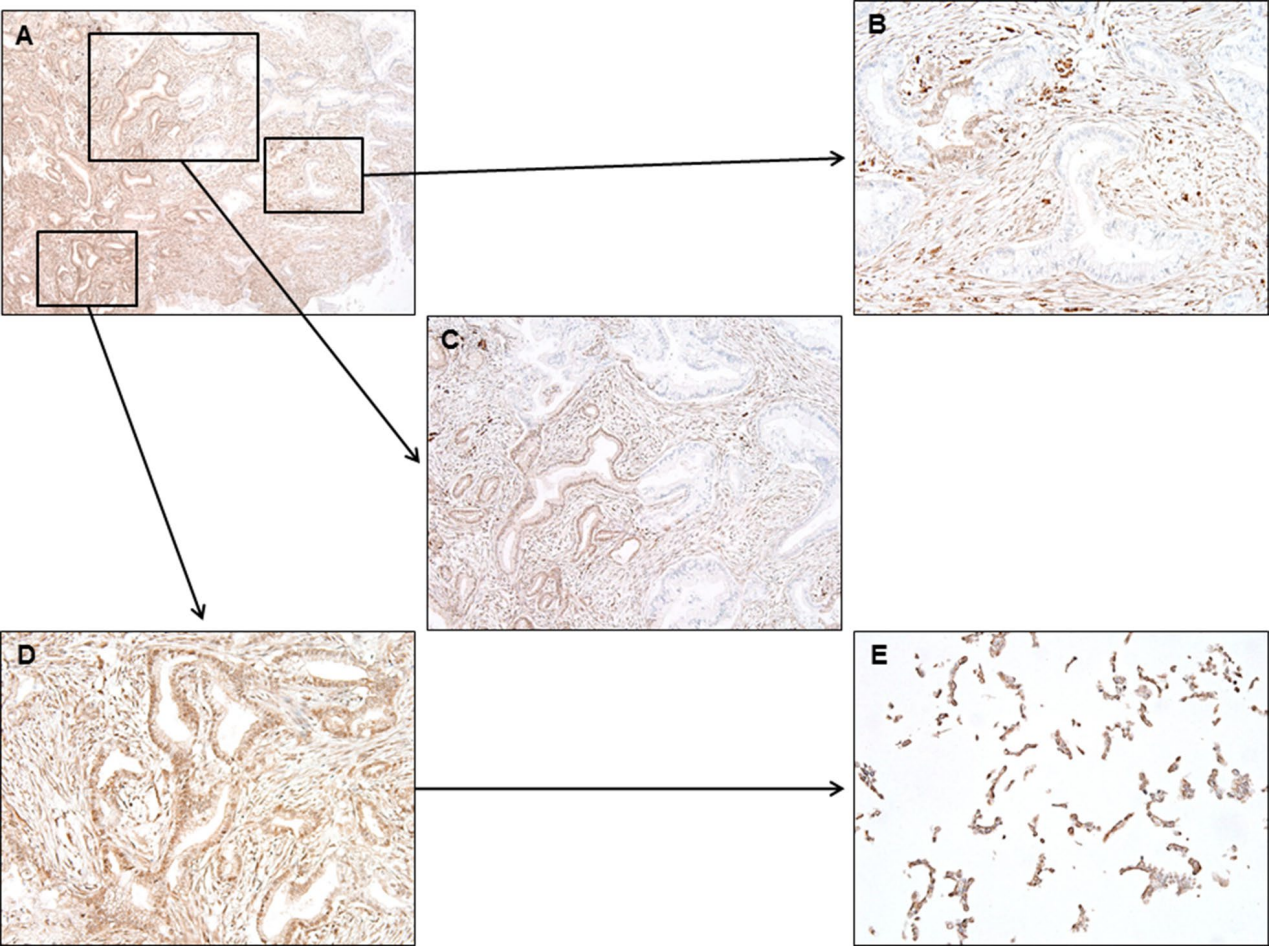
Original cancer tissue of AMCPAC04 showed heterogeneous DPC4 expression. **A** Low-power scanning view shows heterogeneous DPC4 expression (magnification, ×40). **B** Higher power view shows area of loss of DPC4 expression (×200). **C** Cancer cells on the *left half* show intact DPC labeling, while cancer cells on the *right half* show loss of DPC4 expression (×100). **D** Higher power view shows area of intact DPC4 expression (×200). **E** Cancer cells in AMCPAC04 cell line show intact DCP4 labeling (×200)

Because DPC4 protein loss is correlated with *SMAD4/DPC4* mutation, immunohistochemical staining for DPC4 protein is used as a diagnostic criteria of pancreatic cancer with lower cost than sequencing of *SMAD4/DPC4* [50]. Loss of DPC4 expression is associated with distant metastasis, epithelial to mesenchymal transition, and treatment failure to local recurrence [51–53]. In the present study, all 6 AMCPAC cell lines showed homogeneously intact DPC4 expression. In contrast, matched cancer tissues showed heterogeneous DPC4 expression: one tumor area exhibited intact DPC4 expression, while another region showed loss of DPC4 protein expression (Fig. 8). Homogeneously intact DPC4 expression in the primary pancreatic cancer cell lines, which were obtained from cancer tissues with intratumoral DPC4 heterogeneity, can be explained by the positive selection of DPC4-expressing cancer cells during primary cell culture or most DPC4 expressing cancer cells presented in cancer tissues specimens at the time of primary cell culture (Fig. 8).

Genetic heterogeneity occurs in various situations, such as between separate cancers with the same histologic subtypes, primary and metastatic cancers from the same individuals, and separate regions from the same cancers [54–58]. Additionally, genetic heterogeneity has been observed in many solid tumors, including renal cell carcinomas, non-small cell lung cancers, and breast cancers [54, 55, 59–61]. The organoid model of pancreatic cancer was recently examined among preclinical models for predicting personalized tailored therapy [22–24]. This model is expected to conserve the tumor microenvironment, including ductal or acinar structures, and reflect tumor heterogeneity. Therefore, to understand interactions in tumor microenvironments or develop personalized medicine, application of the organoid model will be more effective than conventional primary cancer cell cultures. However, the traditional primary cancer cell culture model is more suitable for investigating the characteristics of pure cancer cells. Nevertheless, methods for establishing specific cell lines by modifying organic models and our culture protocols should be examined in future studies.

AMCPAC cell lines can provide a resource for pancreatic cancer studies, including the basic and translational research fields. For example, comparison of DPC4(+)/P53(−) (AMCPAC01) and DPC4(+)/P53(+) (AMCPAC02–06) cell lines will be helpful in understanding the interactions of *TP53* and *DPC4* signaling pathways. In total, 3 cell lines have been previously established from Korean pancreatic cancer patients, including SNU213, SNU324, and SNU410, which are available from the Korean Cell Line Bank. We have added 6 newly established pancreatic cancer cell lines from Korean patients. Different molecular pathologic characteristics of our new established AMCPAC cell lines may provide diversity and help in determining the molecular pathological basis of pancreatic cancer in different ethnicities from other previously established pancreatic cancer cell lines.

## Conclusions

We established and characterized 6 novel pancreatic cancer cell lines (AMCPAC01–AMCPAC06). These novel cell lines may contribute to the understanding of pancreatic ductal adenocarcinomas, including the molecular basis of tumorigenesis, progression, and metastasis of pancreatic cancers.

## Additional file

**Additional file 1: Figure S1.** *TP53* histogram of AMCPAC cell lines.

## Abbreviations

CMA: cell microarray
FFPE: formalin-fixed paraffin-embedded
