# Supplementary figures and images for: Establishment and characterization of 6 novel patient-derived primary pancreatic ductal adenocarcinoma cell lines from Korean pancreatic cancer patients
Source: Cancer Cell Int. 2017 Apr 20;17:47. doi: 10.1186/s12935-017-0416-8 (PMC5397831; doi:10.1186/s12935-017-0416-8)

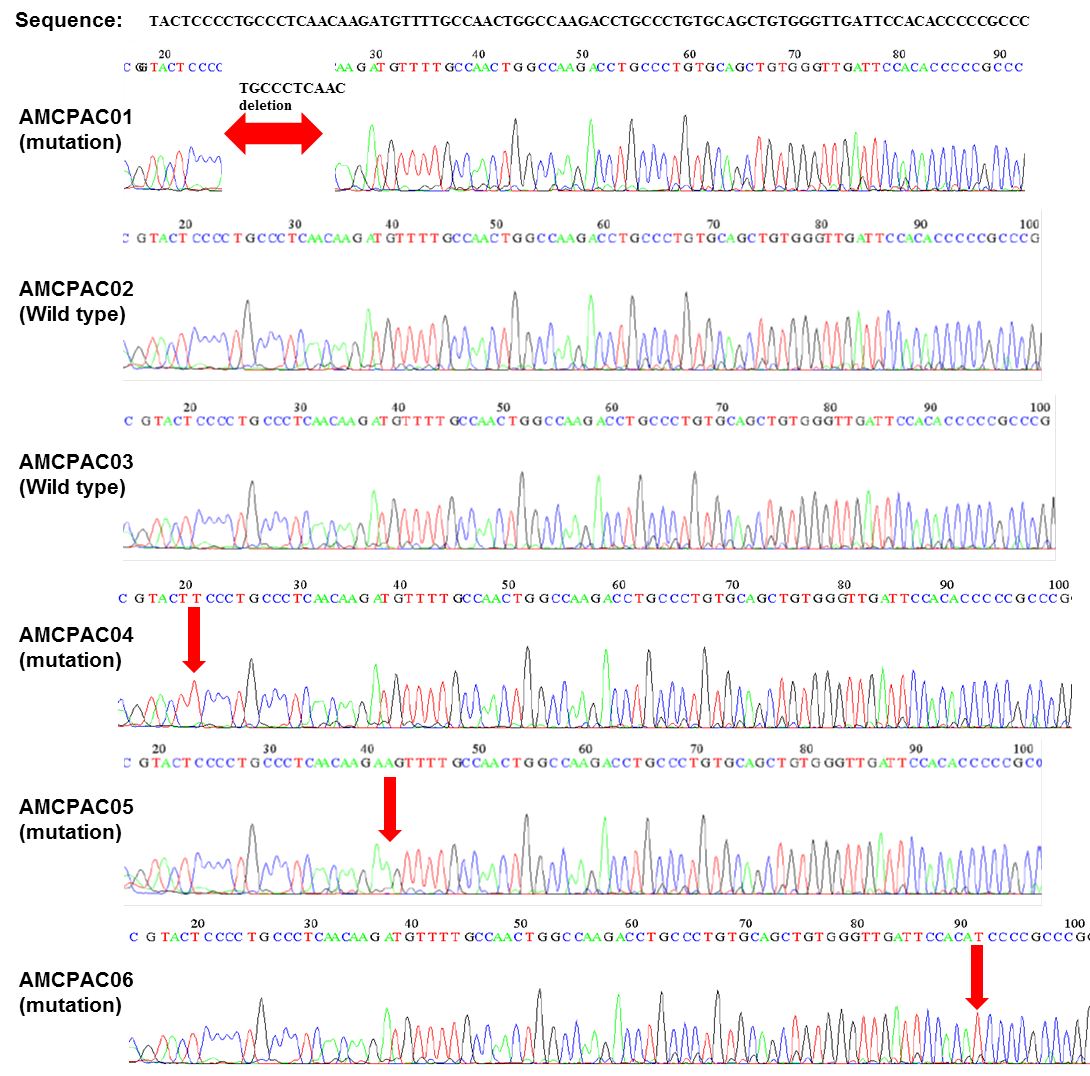

Supplement: Supplementary file 1 — Additional file 1: Figure S1. TP53 histogram of AMCPAC cell lines. [file 12935_2017_416_MOESM1_ESM.tif]
